# Position-locking of volatile reaction products by atmosphere and capping layers slows down photodecomposition of methylammonium lead triiodide perovskite†

**DOI:** 10.1039/d0ra03572f

**Published:** 2020-05-06

**Authors:** Fengshuo Zu, Thorsten Schultz, Christian M. Wolff, Dongguen Shin, Lennart Frohloff, Dieter Neher, Patrick Amsalem, Norbert Koch

**Affiliations:** Institut für Physik & IRIS Adlershof, Humboldt-Universität zu Berlin 12489 Berlin Germany norbert.koch@physik.hu-berlin.de; Institut für Physik und Astronomie, Universität Potsdam 14776 Potsdam Germany; Helmholtz-Zentrum Berlin für Materialien und Energie GmbH 12489 Berlin Germany

## Abstract

The remarkable progress of metal halide perovskites in photovoltaics has led to the power conversion efficiency approaching 26%. However, practical applications of perovskite-based solar cells are challenged by the stability issues, of which the most critical one is photo-induced degradation. Bare CH_3_NH_3_PbI_3_ perovskite films are known to decompose rapidly, with methylammonium and iodine as volatile species and residual solid PbI_2_ and metallic Pb, under vacuum under white light illumination, on the timescale of minutes. We find, in agreement with previous work, that the degradation is non-uniform and proceeds predominantly from the surface, and that illumination under N_2_ and ambient air (relative humidity 20%) does not induce substantial degradation even after several hours. Yet, in all cases the release of iodine from the perovskite surface is directly identified by X-ray photoelectron spectroscopy. This goes in hand with a loss of organic cations and the formation of metallic Pb. When CH_3_NH_3_PbI_3_ films are covered with a few nm thick organic capping layer, either charge selective or non-selective, the rapid photodecomposition process under ultrahigh vacuum is reduced by more than one order of magnitude, and becomes similar in timescale to that under N_2_ or air. We conclude that the light-induced decomposition reaction of CH_3_NH_3_PbI_3_, leading to volatile methylammonium and iodine, is largely reversible as long as these products are restrained from leaving the surface. This is readily achieved by ambient atmospheric pressure, as well as a thin organic capping layer even under ultrahigh vacuum. In addition to explaining the impact of gas pressure on the stability of this perovskite, our results indicate that covalently “locking” the position of perovskite components at the surface or an interface should enhance the overall photostability.

## Abstract 2

Gas pressure and capping layers under ultrahigh vacuum prevent methylammonium lead triiodide photo-degradation due to efficient back-reaction of volatile compounds.

## Graphical abstract

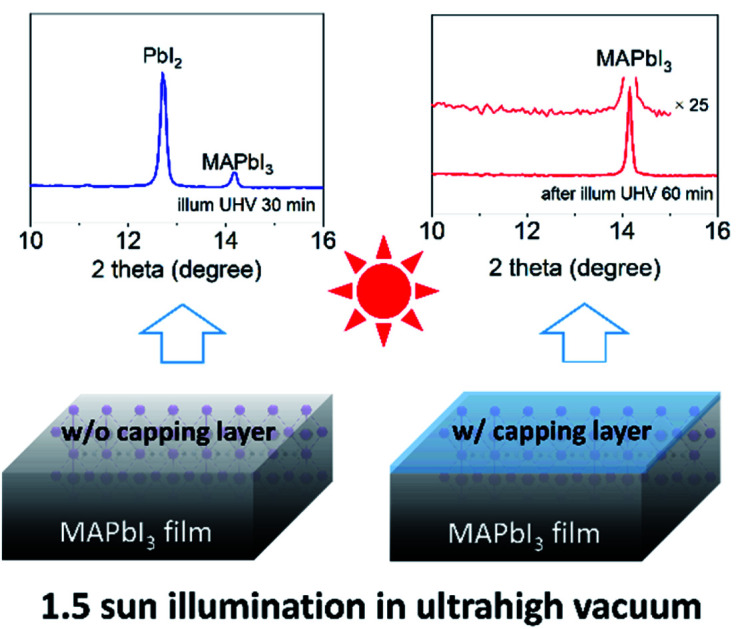

## 1 Introduction

Metal halide perovskites, owing to their remarkable optoelectronic characteristics^1–4^ have shown great potential not only in photovoltaic applications^5–8^ but also in light-emitting devices^9,10^ and photodetectors.^11–13^ In particular, perovskite solar cells have achieved tremendous accomplishments over the past decade, reaching a power conversion efficiency of over 25% for single junction cells.^14^ On the other hand, halide perovskite materials suffer from stability issues,^15–25^ of which the most critical one is that perovskites tend to degrade upon light exposure, thus impeding commercialization. Numerous studies have shown that the light-induced degradation process can be assisted by other factors, such as moisture,^22,24^ oxygen,^22^ an applied electric field,^21^ or simply vacuum.^18,23^ As pointed out by Tang *et al.*^18^ photo-induced degradation is found to be dependent on the environmental atmosphere, where the degradation upon illumination is significantly accelerated under vacuum. In addition, a recent study by Lin *et al.*^26^ found that an excess of charge carriers in the perovskite film upon photoexcitation can accelerate degradation, raising concerns about the materials' intrinsic stability. Nonetheless, it appears crucial to recognize the origin of instabilities of perovskites, especially in different environmental conditions and gas pressure. Therefore, further extensive investigations are needed towards a comprehensive understanding of the intrinsic degradation mechanisms of perovskite materials.

In a previous study,^23^ we demonstrated that upon white light illumination the methylammonium lead mixed halide perovskites (MAPbI_*x*_Cl_3−*x*_, MA: CH_3_NH_3_^+^) degrade rapidly under ultrahigh vacuum (UHV) conditions. Similar observations were also reported by Tang *et al.*,^18^ Juarez-Perez *et al.*,^20^ and Xu *et al.*^25^ who found that vacuum accelerates the photodecomposition process of perovskite films. However, the mechanism accountable for such a rapid photodecomposition process in vacuum has not been adequately explained yet. To provide an overall picture of the light-induced degradation mechanisms in different conditions of pressure and atmosphere, particularly the mechanism relating to the fast degradation process in vacuum, here we applied several complementary experimental techniques to study the optical, chemical, and electronic properties of the archetypical MAPbI_3_ perovskite, while exposing it to light in UHV, as well as under N_2_ and ambient air at atmospheric pressure. We demonstrate that upon white light illumination the rapid decomposition process of MAPbI_3_ into PbI_2_ and metallic Pb in UHV starts predominantly at the surface, resulting in significant changes in the structure and optoelectronic properties. In contrast, illumination in N_2_ and air does not induce substantial degradation even on the timescale of hours. In all cases, yet, we directly evidence the release of iodine from the perovskite surface as the cause for the instability of the perovskite upon photoexcitation. In addition, by employing charge selective and non-selective organic capping layers, the fast photodecomposition process in UHV can be tremendously reduced by restraining the volatile methylammonium and iodine from leaving the surface, indicating the effect of surrounding gas pressure being one of the key factors contributing to stability. Note that capping layers in ambient conditions were already shown to enhance perovskite stability, mainly by inhibiting the contact to water.^27^ While certainly great care must be taken when characterizing bare perovskite materials under (ultrahigh) vacuum conditions, our results also suggest that the back-reaction of volatile degradation products, when kept in proximity, to the parent perovskite is very efficient.

## 2 Results and discussion

### 2.1 Impact of environmental conditions on the photodecomposition process

**Fig. 1:**
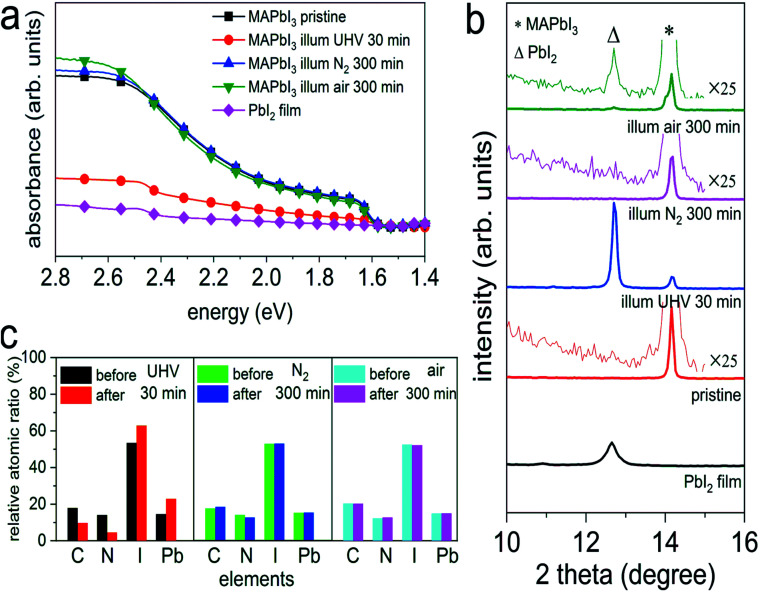
(a) UV-vis absorption spectra of a PbI_2_ film (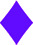), the pristine MAPbI_3_ perovskite films before (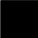), after illumination treatment in UHV for 30 min (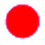), in N_2_ (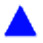), and ambient air (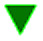) for 300 min, respectively. (b) X-ray diffraction patterns of a PbI_2_ film, and the MAPbI_3_ perovskite films before and after illumination in different environmental conditions. The magnified insets highlight the emergence of the PbI_2_ peak. Symbols Δ and * indicate the peak positions of PbI_2_ (001) and MAPbI_3_ (110), respectively. (c) Evolution of the stoichiometry (exclusive of the metallic Pb contributions) of the same set of perovskite films before and after illumination in different environmental conditions. The corresponding XPS spectra can be seen in Fig. S2–S4 of the ESI.† Above films were deposited on ITO/PEDOT:PSS substrates.

To elucidate the effect of environmental conditions, with a focus on atmospheric pressure *versus* vacuum, on the photo-induced degradation process, MAPbI_3_ films prepared on poly(3,4-ethylenedioxythiophene):poly(styrenesulfonate) (PEDOT:PSS) substrates were exposed to white light (intensity equivalent to *ca.* 1.5 sun, with negligible UV spectral contribution below 400 nm) in UHV (base pressure of 5 × 10^−9^ mbar), as well as in atmospheric pressure of N_2_ and ambient air, respectively, while a variety of characterization techniques were applied to observe changes in the optical, chemical, morphological, and electronic properties. As displayed in Fig. 1a, upon illumination in UHV for 30 min we observe a drastic decrease of the optical absorption over the entire visible range, accompanied by an emergence of an absorption feature at *ca.* 2.5 eV. Such feature corresponds to the absorption of PbI_2_ and confirms the formation of PbI_2_, as also noted by other recent studies.^18,20,25^ In contrast to the case of UHV, illumination in N_2_ and in ambient air, even over extended time (up to 300 min), does not induce noticeable changes in the absorption spectra of Fig. 1a. To further investigate the effect of illumination on the chemical and structural properties, X-ray photoelectron spectroscopy (XPS) and X-ray diffraction (XRD) measurements were performed before and after different illumination treatments. As depicted in Fig. 1b, XRD patterns of all MAPbI_3_ films reveal a diffraction peak at 14.2°, which corresponds to the (110) reflection of the perovskite tetragonal structure [PDF 01-084-7607 (ICDD, 2018)]. The survey scan of the diffraction patterns can be found in Fig. S1 in the ESI.† Upon illumination in UHV one additional diffraction peak at 12.7° appears, which is assigned to the (001) reflection of the PbI_2_ hexagonal structure [PDF 00-007-0235 (ICDD, 1957)]. The appearance of a strong PbI_2_ diffraction peak indicates a severe photo-induced degradation in UHV condition, in agreement with the optical absorption results. In comparison, illumination in N_2_, even for a period of 300 min, does not induce noticeable formation of PbI_2_. We note that, however, illumination in ambient air produces a trace of PbI_2_, as shown in the magnified insets in Fig. 1b. Such a minor and rather slow photodecomposition process in ambient air is most likely assisted by the presence of water, as found in recent studies.^18,22^

In addition to the structural changes, analysis of the evolution of the stoichiometry by XPS consistently reveals in an analogous manner that the composition of the MAPbI_3_ films has been largely modified upon light exposure in UHV, as illustrated in Fig. 1c. We observe severe loss of carbon and nitrogen, as well as a decrease of the relative iodine content by 40%, accompanied by the formation of a large amount of metallic Pb (Pb^0^) on the surface; further discussed in Fig. S2–S4.† Note that the seemingly increased iodine content in Fig. 1c is due to the fact that the sum of relative carbon, nitrogen, iodine, and lead components is 100% for each sample (only the metallic Pb amount is excluded in the atomic ratios), while the decrease of iodine content is estimated from the change in the absolute photoelectron intensity of iodine 3d_5/2_ peaks before and after illumination. In addition, angle-dependent XPS measurements, as presented in Fig. S5,† exhibit strongly varying Pb^0^/Pb^2+^ ratios (from 0.9 to 1.8) when changing the emission angle from 0° (normal emission) to 80°. Since XPS is a very surface sensitive method (probing depth of few nm) due to the short mean free path of photoelectrons,^28^ a change of the emission angle to off-normal enhances surface sensitivity even more. The variation of Pb^0^/Pb^2+^ ratios that we observe here thus indicates a vast enrichment of Pb^0^ at the surface, suggesting that the photodecomposition process proceeds from the surface on and does not occur homogeneously throughout the bulk. Consistent with the optical and structural changes induced by illumination in N_2_ and ambient air, we do not observe notable variations in the stoichiometry before and after illumination treatments, as seen in Fig. 1c. Furthermore, atomic force microscope (AFM) measurements, as displayed in Fig. S6,† show that the surface morphology is significantly modified after illumination in UHV, while the topographic features of the N_2_- and air-illuminated perovskite films remain very similar to those of the pristine samples. Specifically, the granular appearance of the film surfaces persists upon illumination, but the increase in surface roughness is much smaller in air and N_2_ (root-mean-square roughness increases from *ca.* 7 nm to *ca.* 9 nm) compared to UHV (roughness of *ca.* 38 nm), where also a few deep holes appear. Above observations confirm previous findings that photo-induced degradation is significantly accelerated in UHV and can be largely reduced in inert atmosphere, in agreement with the studies by Tang *et al.*^18^ and Juarez-Perez *et al.*^20^

To gain further insight into the photodecomposition process of the perovskite in the different environments, we further mounted an indium tin oxide (ITO)/glass coupon about 1 mm above the perovskite samples during light exposure, as illustrated in Fig. S7.† This ITO “cover” is used to “capture” volatile reaction products leaving the perovskite surface, to then be analyzed by XPS. Firstly, it is worth noting that in all environmental conditions we detected iodine on the ITO covers. This directly confirms the release of iodine from the perovskite films, as also found by Zhang *et al.*,^29^ Kim *et al.*,^15^ and Juarez-Perez *et al.*^20^ Furthermore, the release of iodine can accelerate the degradation process *via* chemical chain reactions, as suggested by Wang *et al.*^16^ In addition, we also noticed a slight increase of pressure during illumination in UHV, from 5 × 10^−9^ mbar to 3 × 10^−8^ mbar, which can be directly related to the release of iodine as well as CH_3_NH_3_^+^, as also concluded from the stoichiometry analysis above.

**Fig. 2:**
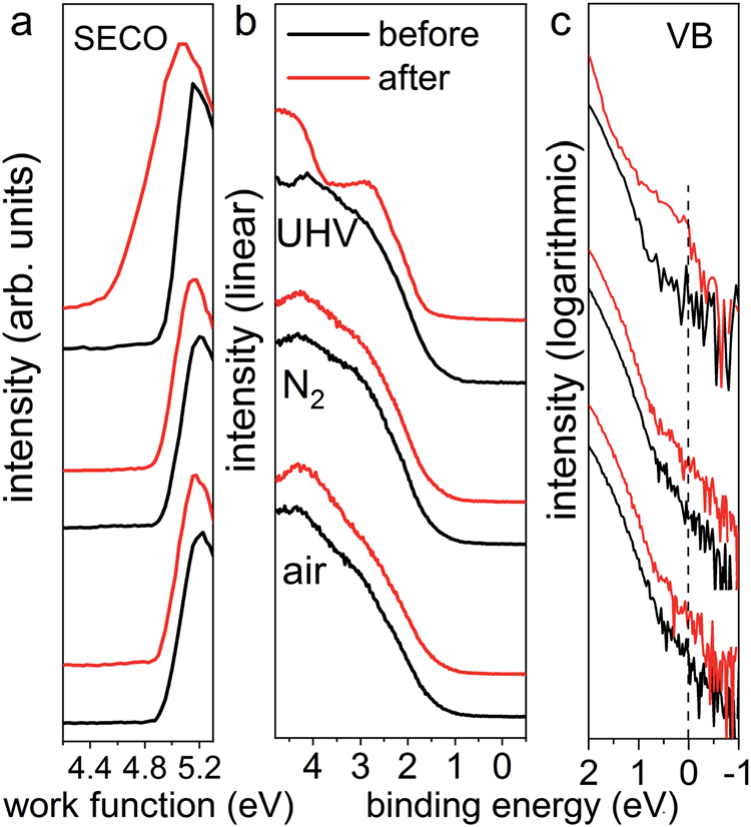
UPS spectra of the same set of MAPbI_3_ perovskite films (from Fig. 1) before and after light exposure in UHV for 30 min, in N_2_ and ambient air for 300 min, respectively. Left panel (a) shows the SECO region, and middle (b) and right (c) panels show the valence band region on linear and logarithmic intensity scale, respectively. The dashed line in the right panel indicates the Fermi level at 0 eV binding energy.

Furthermore, the impact of illumination on the electronic structure was examined by ultraviolet photoelectron spectroscopy (UPS), as displayed in Fig. 2. Here, all pristine perovskite films show nearly identical work function values of 4.94 ± 0.01 eV, as seen from the secondary electron cut-off (SECO) region, and exhibit highly reproducible valence band structures. As pointed out by Endres *et al.*^30^ and in our recent study,^31^ due to the very low density of states (DOS) from the highly dispersive top valence bands (VB), an accurate value of the VB onset can be achieved only on a logarithmic intensity scale, while values determined on a linear intensity scale overestimate the VB onset. Therefore, the valence band spectra are also presented on logarithmic scale, as shown in Fig. 2c, from which almost identical onset values of 0.80 ± 0.04 eV are observed for all pristine samples. After illumination in UHV, we observe that the valence band structure changes significantly, giving rise to noticeable DOS across the Fermi level (binding energy at 0 eV), as clearly seen in logarithmic scale in Fig. 2c. This DOS is ascribed to the high abundance of metallic Pb on the surface, as shown in Fig. S2,† which acts as donor-type surface states. Concomitantly, as seen from the SECO region, the work function decreased from 4.94 eV to 4.55 eV as a result of downward band bending induced by the Pb^0^-related donor-type surface states. In contrast and as expected, the UPS data for the illumination treated films in N_2_ and ambient air exhibit essentially the same SECO and VB spectral shape and energy position before and after light exposure. These UPS results suggest that, although the optical properties and electronic structure of the N_2_- and air-illuminated perovskite films remain mostly unchanged before and after light exposure, the degradation process has already commenced, as evidenced by the release of some iodine (from XPS measurements on the ITO covers). In short, we find that the photodecomposition process of MAPbI_3_ perovskite films highly depends on the environmental conditions, where fast degradation of MAPbI_3_ into solid PbI_2_ and metallic Pb and volatile methylammonium and iodine, in UHV condition occurs. This is in stark contrast to the situation in N_2_ and ambient air at atmospheric pressure, in which the photo-induced degradation is tremendously slowed down, in particular under the inert gas.

### 2.2 Impact of organic capping layers on the photodecomposition process

#### 2.2.1 Charge carrier non-selective organic capping layer

**Fig. 3:**
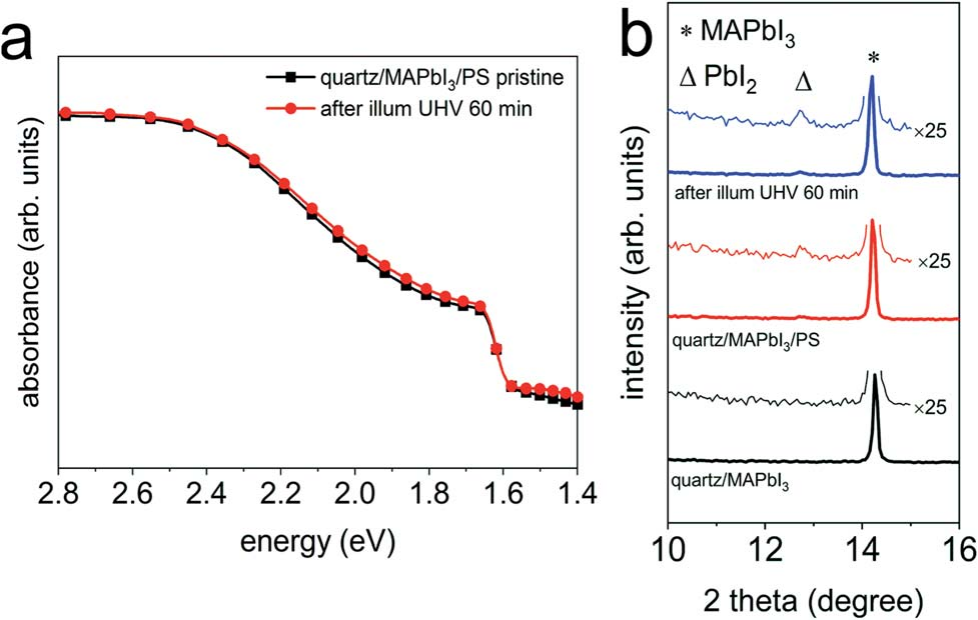
(a) UV-vis absorption spectra of a quartz/MAPbI_3_/PS film before (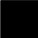) and after light exposure (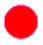) in UHV for 60 min. (b) XRD patterns of the same set of samples before and after light exposure in UHV for 60 min, together with a pristine quartz/MAPbI_3_ film as a reference sample. Wide range XRD patterns are shown in Fig. S8a.† Symbols Δ and * indicate the peak position of PbI_2_ (001) and MAPbI_3_ (110), respectively.

To understand the origin of the accelerated photodecomposition process in vacuum, we first investigate the impact of a charge carrier non-selective capping layer on the photo-induced degradation process of MAPbI_3_, as also suggested by Tang *et al.*^18^ To this end, MAPbI_3_ films were fabricated on quartz substrates, which were further covered by a thin (*ca.* 50 nm) insulating layer of polystyrene (PS). Such samples, with the configuration quartz/MAPbI_3_/PS, were illuminated with the same light intensity (*ca.* 1.5 sun) in UHV over a period of 60 min. Fig. 3a shows the corresponding optical absorption spectra. Clearly, after 60 min light exposure, the film exhibits essentially identical absorption features as the pristine film, indicating effective suppression of the MAPbI_3_ decomposition. This results from a change in the equilibrium conditions of the MAPbI_3_ surface when interfaced with vacuum or with another solid, at the otherwise same conditions of outside pressure (UHV), temperature, and light exposure (1.5 sun). XRD diffraction patterns of the pristine quartz/MAPbI_3_, pristine quartz/MAPbI_3_/PS, and illuminated quartz/MAPbI_3_/PS samples show essentially the same diffractograms, confirming the fact that neither chemical nor structural properties are changed. We note that a barely discernible PbI_2_ peak arises already after spin-coating of the PS layer and is consistently ascribed to the processing of MAPbI_3_ with the PS solution; the intensity of the PbI_2_ peak barely increases upon light illumination. Overall, these findings further substantiate that the MAPbI_3_ degradation occurs from the film surface onwards, and one effect of the capping layer is clearly to prevent volatile degradation reaction products from leaving the surface.

Based on the above results, we thus propose the following photodecomposition reaction, where we explicitly consider the reaction rate for photo-induced degradation (*r*_D_) and that for the back-reaction to the parent perovskite (*r*_B_):
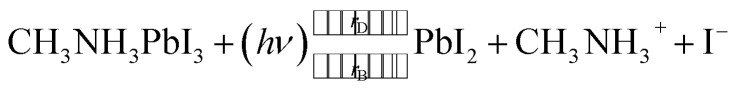


Upon photon (*hν*) absorption (which is part of above equation only for the degradation reaction, not for the back-reaction), volatile CH_3_NH_3_^+^ and I^−^ ions are generated, and PbI_2_ may exist in an intermediate configuration. In vacuum, the volatile products are released from the surface, leaving behind (intermediate) PbI_2_. Subsequently, PbI_2_ can be decomposed into Pb^0^ and I_2_*via* a photolysis reaction process.^18^ This photodegradation reaction can certainly take place also in the bulk, but since the reaction products remain in immediate vicinity the back-reaction can proceed with high rate, *i.e.*, *r*_D_ and *r*_B_ are very similar, and photodegradation in the bulk is very slow overall. In contrast, the volatile degradation products can readily leave the surface in vacuum and *r*_B_ approaches zero, resulting in continued decomposition and material loss. It is readily plausible that a solid capping layer, such as the PS layer in our experiments, reduces the loss rate of reaction products from the perovskite surface and thus enables the back-reaction rate *r*_*B*_ to become large. Apparent from our experiments, the effect of the solid capping layer on the photodegradation kinetics is very similar to that of N_2_ and air at atmospheric pressure. The high collision rate of gas molecules (*e.g.*, while at a pressure of 10^−6^ mbar it takes approx. 1 s that every surface site is hit by a gas atom, it takes only approx. 1 ns at atmospheric pressure) with the perovskite surface, and thus also the reaction products formed upon the photo-induced reaction, is sufficient to keep the volatile ions in close proximity on a time scale such that again *r*_*D*_ ≈ *r*_*B*_. In turn, the macroscopically observed degradation process, yet ensuing from the solid surface onwards, is significantly slowed down compared to vacuum as environment.

#### 2.2.2 Charge-carrier selective organic capping layer

**Fig. 4:**
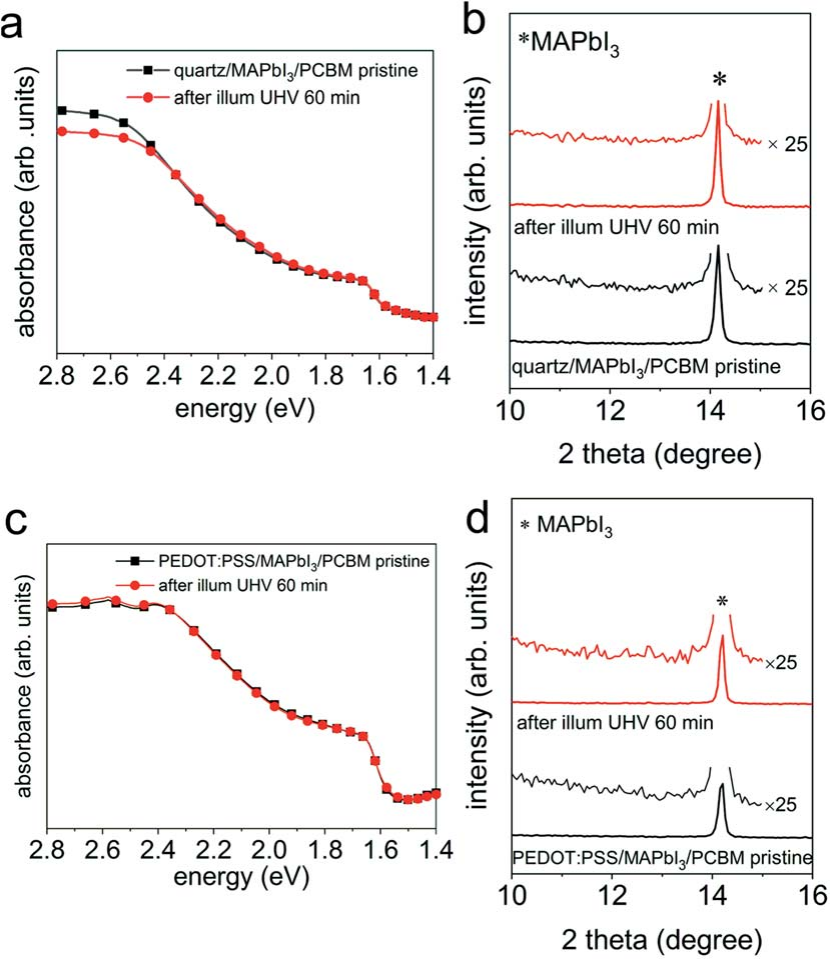
UV-vis absorption spectra of (a) a quartz/MAPbI_3_/PCBM sample and (c) a PEDOT:PSS/MAPbI_3_/PCBM sample before (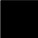) and after (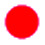) light exposure in UHV for 60 min. (b) and (d) XRD patterns of the same set of samples from UV-vis absorption measurements. Symbol * indicates the (110) peak position of MAPbI_3_.

Recent investigations on the photodecomposition mechanisms of perovskite materials also suggested that an excess of free charges, and holes in particular, can accelerate the degradation process.^26^ To unravel whether such an effect plays a role under the conditions of our studies above, the same illumination experiments were performed with a sample configuration of quartz/MAPbI_3_/PCBM (with *ca.* 6 nm PCBM), where PCBM stands for [6,6]-phenyl-C61-butyric acid methyl ester, which is a prototypical electron transport material in perovskite solar cells. Under steady-state illumination, electron accumulation in PCBM occurs, and correspondingly a certain excess of holes, of yet unknown magnitude and spatial distribution, in the perovskite layer is expected. As shown in Fig. 4a (wide range XRD patterns can be found in Fig. S8b†), the absorption spectrum of such a sample structure shows essentially the same features in the visible range before and after illumination in UHV for 60 min. The minor changes at energies above 2.5 eV are attributed to a slight canting of the reference bare substrate in the beam path of the spectrometer. The XRD patterns, as displayed in Fig. 4b, do not exhibit any noticeable structural change of the illuminated perovskite sample and no PbI_2_ formation, thus suggesting a proper stability of the perovskite with an electron extracting (PCBM) capping layer, similar to what we found for the electronically inert insulator PS above.

To further investigate this type of “electronically active” interface between MAPI_3_ and PCBM, we also fabricated a sample structure consisting of ITO/PEDOT:PSS/MAPbI_3_/PCBM, such that the density of the excess charge carriers is reduced in the perovskite layer upon photoexcitation, as a result of charge extraction and interface-mediated recombination at both interfaces.^32,33^ Analogous to the above results without hole extraction layer, we do not observe any change in the optical properties and structure before and after light exposure in UHV for 60 min, as seen from the absorption spectra in Fig. 4c and the XRD patterns in Fig. 4d.

**Fig. 5:**
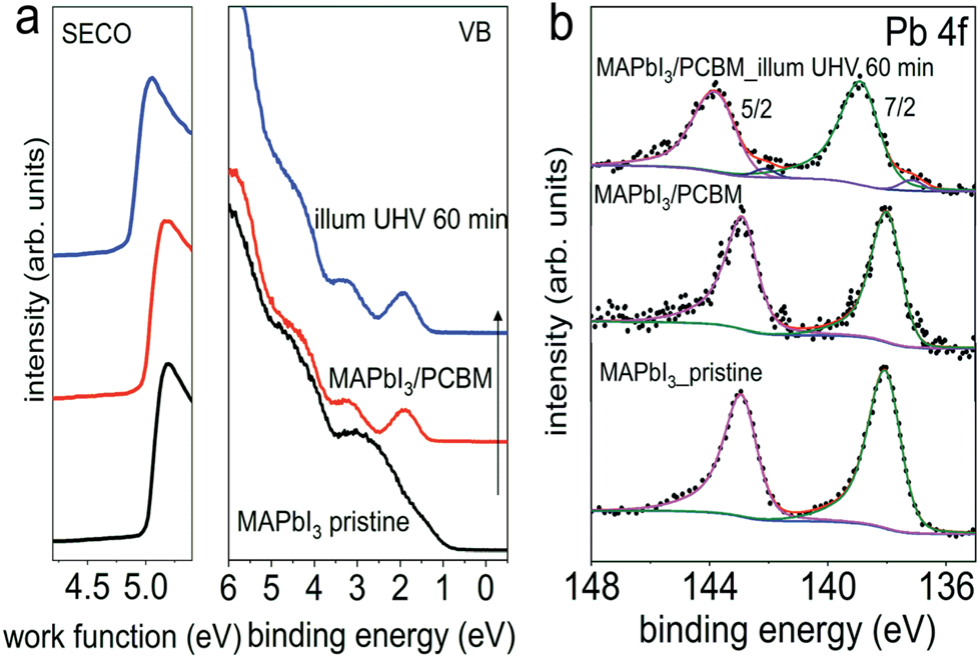
Evolution of the UPS (a) and Pb 4f core levels spectra (b) of a pristine PEDOT:PSS/MAPbI_3_ sample upon deposition of a thin layer of PCBM, and subsequent light exposure in UHV for 60 min.

However, these measurements are not sensitive to eventual minute changes at the very interface between MAPI_3_ and PCBM. Therefore, in addition, UPS and XPS measurements on the same set of samples with the configuration of ITO/PEDOT:PSS/MAPbI_3_/PCBM from Fig. 4, before and after light exposure in UHV were performed sequentially, and the corresponding spectra are shown in Fig. 5. Starting with a work function of 5.00 eV for the pristine perovskite film, essentially vacuum level alignment persists upon deposition of a thin PCBM layer (*ca.* 6 nm), as the complete heterostructure exhibits a work function of 4.98 eV. At this point we mention that a linear extrapolation on linear intensity scale of the photoemission onset is applied for the molecular semiconductors PCBM owing to its non-dispersive and Gaussian-shaped DOS.^34^ Therefore, the VB onset is found at 0.70 eV on a logarithmic intensity scale for the pristine perovskite film, and the onset of the top-layer PCBM highest occupied molecular orbital (HOMO) level at 1.30 eV on a linear intensity scale. The corresponding energy level diagrams are depicted in Fig. S9,† exhibiting a type II heterojunction. Consistent with initial interfacial vacuum level alignment, the Pb 4f peaks of MAPI_3_ remain at constant binding energy upon overcoating with PCBM. After visible light (*ca.* 1.5 sun) exposure in UHV for 60 min, however, we notice a very small amount of metallic Pb formed at the interface, as seen in the corresponding Pb 4f core level spectrum in Fig. 5b. This formation of Pb^0^ is accompanied by a release of elemental iodine, as evidenced by XPS measurements shown in Fig. S10,† which was obtained with the “ITO cover” technique described above. Apparently, the *ca.* 6 nm thin PCBM layer (deduced from the intensity attenuation of Pb 4f core levels) still enabled diffusion of the volatile iodine photo-induced degradation product and its loss through the sample surface. Such metallic Pb^0^, although at a very low density (Pb^0^/Pb^2+^ of 4.2%), is expected to act as donor-type surface state. Accordingly, we do observe a shift of the perovskite-related Pb 4f core level by 0.9 eV towards higher binding energy, as expected from the concomitant downward surface band bending in MAPI_3_ due to these surface states. As a side note, the presence of Pb^0^-related defects, mostly on the surface as depicted in Fig. S5,† could potentially enhance non-radiative interfacial recombination, which thereby degrades solar cell performance. Remarkably, the HOMO level of PCBM remains at the same binding energy after light exposure, which we attribute to the pinning of the lowest unoccupied molecular orbital (LUMO) level to the Pb^0^-related surface states, as schematically shown in the energy level diagram in Fig. S9b.† Consequently, the energy level alignment between the perovskite and the PCBM is markedly modified by illumination with visible light. Thus it is important to note that, while no obvious changes of chemical and structural properties from optical and XRD results were apparent, the electronic properties of the interface are much more sensitive to even minute compositional changes. If they go unnoticed, any correlation between assumed interfacial energy levels and device performance could be significantly flawed.

We note that the sample temperature can rise up to 55 °C during the illumination in our experiments. As demonstrated by previous studies,^23,35^ a mild annealing up to 54 °C does not induce noticeable changes in the chemical and electronic structures of the perovskite films. However, the increased temperature during illumination can possibly accelerate the photodecomposition process, as pointed out by Misra *et al.*^36^

Finally, the above results substantiate that the rapid photodecomposition of MAPI_3_ in UHV can be significantly slowed down by depositing a solid organic capping layer onto the perovskite surface, because the degradation proceeds mainly from the surface onwards and not homogeneously throughout the bulk. The primary effect of the capping layer is to restrain the mobile CH_3_NH_3_^+^ and I^−^ ions from escaping into vacuum, which, as a result, helps balancing the rates of the photo-induced decomposition reaction *r*_D_ and the back-reaction to the parent perovskite *r*_B_. Notably, we conclude that surrounding gas at atmospheric pressure has the same effect in terms of improving the material's stability against photodegradation.

## 3 Conclusions

In summary, using complimentary experimental techniques we have shown that the photodecomposition process of MAPbI_3_ perovskite films is dominated by the loss of volatile reaction products (the methylammonium and iodine) from the surface, and that photo-induced degradation in the bulk plays a minor role. This loss is highly efficient in (ultrahigh) vacuum and leaves behind PbI_2_ and metallic Pb. The photo-induced degradation process can be significantly slowed down (much more than an order of magnitude) by exposing the perovskite surface to N_2_ or simply ambient air at atmospheric pressure, because the frequent collisions of gas atoms/molecules with the surface facilitate that the reaction products remain in close proximity for a prolonged time. This, in turn, enables the back-reaction to the parent perovskite to proceed with high effectiveness. A solid capping layer on top of the perovskite surface has a fully analogous effect. Notably, we do not find significant differences in the photo-induced decomposition kinetics for charge selective and non-selective capping layers. Yet, the back-reaction will not reach 100% and diffusion of reaction products through the capping layer will, over time, lead to complete loss of the perovskite. However, light-induced changes (due to perovskite photodegradation) in the electronic structure of interfaces between the perovskite and charge transport layers can occur way before significant decomposition could be noticed by common analytical methods. For the example of MAPI_3_ and PCBM, our photoemission results evidence that only 30 min illumination of the interface with 1.5 sun white light the interfacial energy level alignment is affected by up to 0.9 eV, a tremendous magnitude in terms of device function. At this stage, optical absorption and XRD measurements did not indicate any degradation yet, but we identified with photoemission experiments interfacial Pb^0^ formation and loss of iodine through the PCBM layer as causes. As noted by numerous others before, great care must be taken when characterizing perovskite materials using high dose photons, particularly in vacuum, as changes in the surface/interface electronic structure also impacts excited state and carrier spatial distribution and dynamics. Given that the surface is most critical for the stability of MAPI_3_, and probably also other perovskites, it should be worthwhile considering methods for covalent “locking” the surface components of the material in place.

## 4 Experimental details

### 4.1 Sample preparation

The MAPbI_3_ perovskite films were prepared following our previous study,^37^ however, the 1 : 1 molar ratio of methylammonium iodide to lead(ii) iodide was changed to 1 : 0.95 in order to obtain a pure perovskite crystal structure without appearance of PbI_2_, as shown in Fig. S11 in the ESI.† Methylammonium iodide (MAI, 99%, anhydrous), lead(ii) iodide (99.999% trace metals basis, beads), *N*,*N*-dimethylformamide (DMF) (99.8%, anhydrous) and diethyl ether (99.7%, anhydrous) were used as received from Sigma-Aldrich.

[6,6]-Phenyl C61 butyric acid methyl ester (PCBM, 99.9% from Sigma-Aldrich) films were fabricated by spin-coating the PCBM precursor (10 mg mL^−1^ in chlorobenzene) at 1000 rpm for 60 s onto the perovskite film. Polystyrene (PS) films were prepared by spin-coating the PS precursor (average molecular weight of 190.000, 10 mg mL^−1^ in 1,2-dichlorobenzene) at 1000 rpm for 60 s. Subsequently, the PCBM and PS films were annealed at 100 °C for 5 min. All films were prepared in the N_2_-filled glove box and transferred directly into the vacuum chamber under N_2_ atmosphere for photoemission measurements.

### 4.2 UV-vis absorption

UV-vis absorption measurements were performed in air using a Lambda 950 spectrometer (PerkinElmer) in transmission mode.

### 4.3 X-ray diffraction

X-ray diffraction measurements were conducted in air using a Bruker D8 Advanced diffractometer at a wavelength of 0.154 nm.

### 4.4 Photoemission spectroscopy

The X-ray and ultraviolet photoelectron spectroscopy (XPS and UPS) measurements shown in Fig. 1 and 2 were conducted at a JEOL JPS-9030 UHV system (base pressure of 1 × 10^−9^ mbar), using monochromatized Al Kα (1486.6 eV) radiation for XPS and hydrogen Lyman α (10.2 eV, Excitech)^38^ for UPS measurements. XPS and UPS experiments shown in Fig. 5 were performed at a SPECS UHV system (base pressure of 5 × 10^−10^ mbar), which is equipped with a monochromatized helium discharge lamp (21.22 eV) for UPS and a standard Mg Kα (1253.6 eV) X-ray source for XPS measurements. The SECO spectra were recorded at a bias of −10 V on the samples to overcome the work function of the analyzer. All spectra were recorded at room temperature and normal emission (unless otherwise stated).

Sample illumination in different environmental conditions was conducted using a white halogen lamp (Solux MR16 4700 K, 50 watt, daylight rendering, the emission spectrum can be obtained at www.solux.net/cgi-bin/tlistore/infopages/4700k.html#TechnicalSpecifications) in UHV (base pressure of 5 × 10^−9^ mbar), in a N_2_-filled glove box (water and oxygen content less than 0.1 ppm), and in ambient air (relative humidity at 20%), respectively, at a controlled intensity of *ca.* 150 mW cm^−2^.

## Author contributions

The project was conceived by F. Z. and N. K. F. Z. performed the characterizations, data analysis and wrote the first manuscript. F. S., D. S., C. W., and L. F. prepared the samples. All authors assisted with interpretation of the results and contributed to the writing of the manuscript.

## Conflicts of interest

There are no conflicts to declare.

## Supplementary Material

RA-010-D0RA03572F-s001
