# Supplementary material for: Position-locking of volatile reaction products by atmosphere and capping layers slows down photodecomposition of methylammonium lead triiodide perovskite
Source: RSC Adv. 2020 May 6;10(30):17534–42. doi: 10.1039/d0ra03572f (PMC9053590; doi:10.1039/d0ra03572f)
Supplement: RA-010-D0RA03572F-s001 [file RA-010-D0RA03572F-s001.pdf]

Electronic Supplementary Material (ESI) for RSC Advances.
This journal is © The Royal Society of Chemistry 2020

Supporting Information for

Position-locking of volatile reaction products by atmosphere and capping layers slows down photodecomposition of methylammonium lead triiodide perovskite

Fengshuo Zu,[a] Thorsten Schultz,[a] Christian M. Wolff,[b] Dongguen Shin,[a] Lennart Frohloff,[a] Dieter Neher,[b] Patrick Amsalem,[a] and Norbert Koch[*a,c]*

[a]Institut für Physik & IRIS Adlershof, Humboldt-Universität zu Berlin, 12489 Berlin, Germany
[b]Institut für Physik und Astronomie, Universität Potsdam, 14776 Potsdam, Germany
[c]Helmholtz-Zentrum Berlin für Materialien und Energie GmbH, 12489 Berlin, Germany

The wide range X-ray diffraction (XRD) measurements of a PbI_2 film and the pristine PEDOT:PSS/$MAPbI_3$ perovskite films before and after illumination treatments in different environmental conditions are shown in Fig S1. The XRD peaks appeared at 14.2°, 20.1°, 23.6°, 24.6° and 28.5° correspond to the (110), (200), (211), (202) and (220) planes of the perovskite tetragonal structure, according the PDF card [PDF 01-084-7607 (ICDD, 2018)]. Peak at 12.7° is assigned to be the (001) plane of the PbI_2 hexagonal structure [PDF 00-007-0235 (ICDD, 1957)].

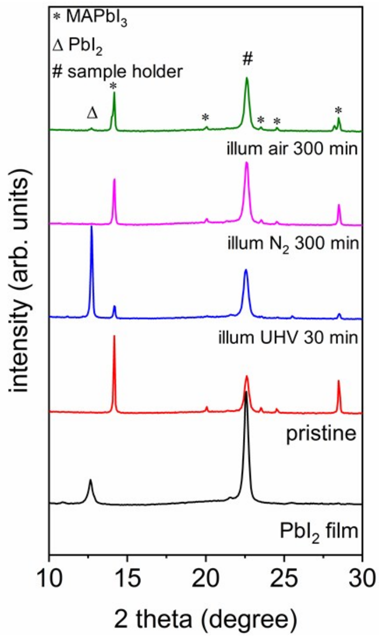

Fig S1. X-ray diffraction patterns of a PbI_2 film, and the pristine $MAPbI_3$ perovskite films before and after illumination treatment in different environmental conditions. Symbol "△" and "*" corresponds the peak positions of PbI_2 and $MAPbI_3$, respectively. PbI_2 (001) peak position accords to PDF 00-007-0235 (ICDD, 1957) and $MAPbI_3$ peaks to PDF 01-084-7607 (ICDD, 2018).

X-ray photoelectron spectroscopy (XPS) spectra of the PEDOT:PSS/$MAPbI_3$ films before and after illumination treatments in UHV, N_2 and ambient air conditions are shown in Fig S2, Fig S3 and Fig S4, respectively. We note that in all cases, C 1s spectra consist of two peaks at 285.8 eV (C_H) and 284.4 eV (C_L) for all pristine perovskite samples with C_H being much higher than C_L in intensity. Here, the C_H peak is assigned to be the $CH_3NH_3^+$ from perovskite structure, and the C_L is possibly due to the presence of CH_3NH_2, according to previous reports.[1,2] Therefore, quantitative analyses of the stoichiometry upon illumination treatments were conducted using the C 1s (peaks of C_H), N 1s, Pb 4f 7/2 and I 3d5/2 peaks, as displayed in Fig 1c.

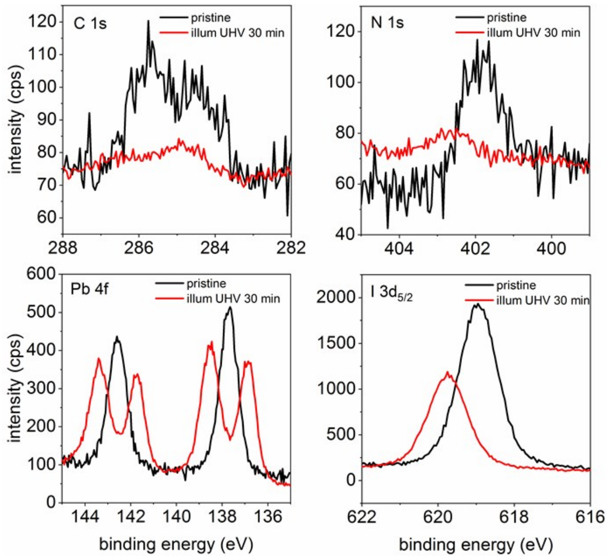

Fig S2. XPS core levels spectra of C 1s, N 1s, Pb 4f and I $3d_{5/2}$ of the PEDOT:PSS/$MAPbI_3$ films before (black line) and after illumination treatment (red line) in UHV condition for 30 min. Spectra were recorded at JPS-9030 UHV system.

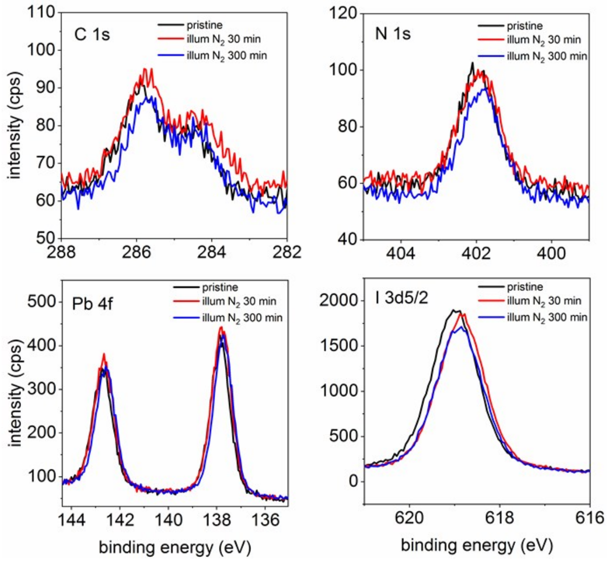

Fig S3. XPS core levels spectra of C 1s, N 1s, Pb 4f and I $3d_{5/2}$ of the PEDOT:PSS/$MAPbI_3$ films before (black line) and after illumination treatment in N_2 condition for 30 min (red line) and 300 min (blue line), respectively. Spectra were recorded at JPS-9030 UHV system.

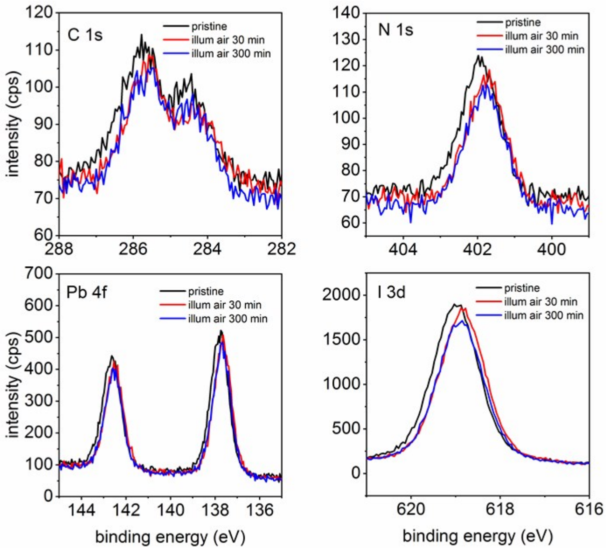

Fig S4. XPS core levels spectra of C 1s, N 1s, Pb 4f and I $3d_{5/2}$ of the PEDOT:PSS/$MAPbI_3$ films before (black line) and after illumination treatment in ambient air condition for 30 min (red line) and 300 min (blue line), respectively. Spectra were recorded at JPS-9030 UHV system.

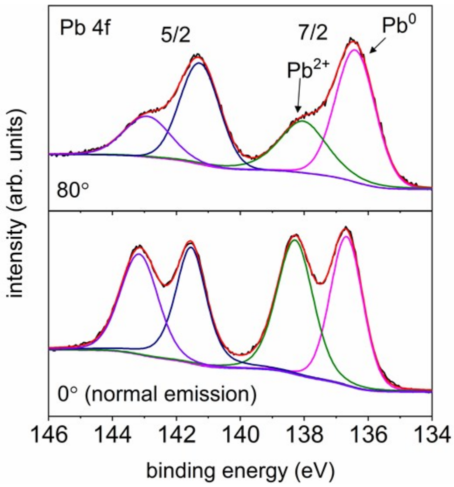

Fig S5. Angle-dependent XPS spectra of the Pb 4f core levels of the PEDOT:PSS/ $MAPbI_3$ films after illumination treatment in UHV condition for 30 min.

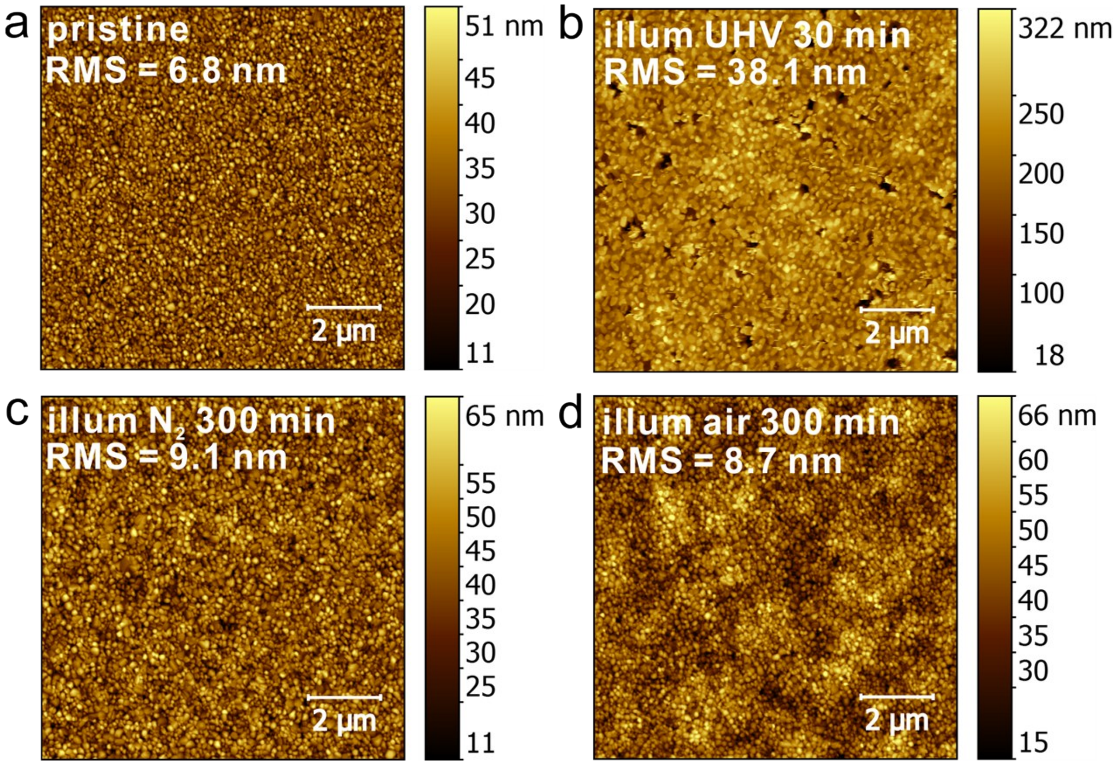

Fig S6. AFM morphology of the (a) pristine PEDOT:PSS/ $MAPbI_3$ films and after illumination treatments in (b) UHV for 30 min, (c) N_2 and (d) ambient air for 300 min.

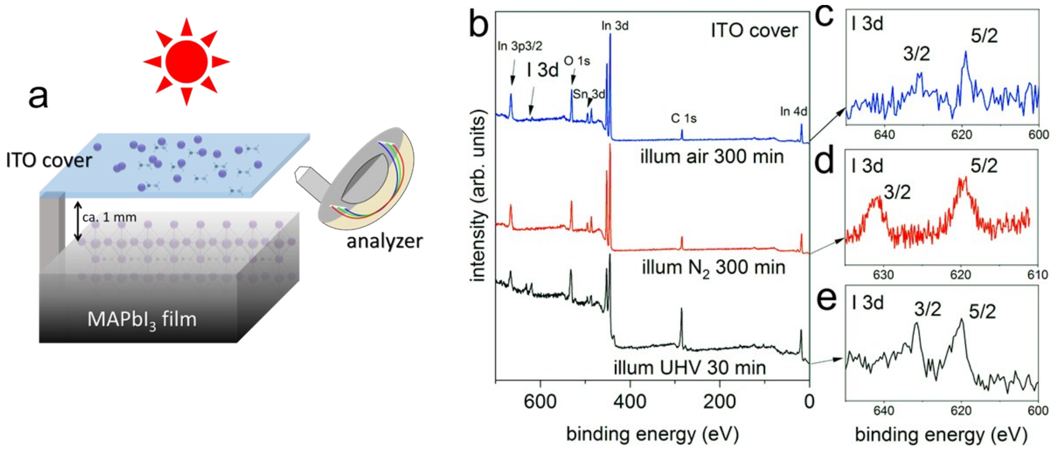

Fig S7. (a) Schematic illustration of ITO covers mounted on top of the perovskite films during illumination process. (b) XPS survey spectra of the ITO covers as illustrated in (a) during illumination treatments in UHV, N_2 and ambient air conditions. (c), (d) and (e) are the magnified spectra of the corresponding ITO covers in UHV, N_2 and air, respectively, in the energy region of iodine.

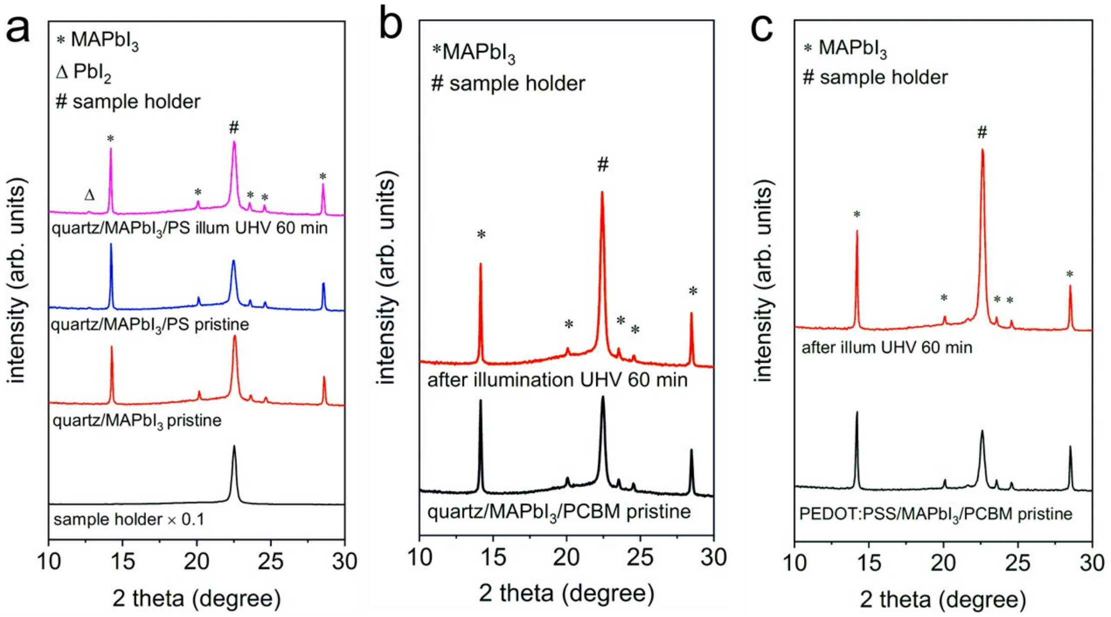

Fig S8. X-ray diffraction patterns of (a) a reference sample holder, the pristine quartz/$MAPbI_3$/PS films, (b) the pristine quartz/$MAPbI_3$/PCBM films and (c) the pristine PEDOT:PSS/$MAPbI_3$/PCBM films before and after illumination treatment in UHV condition for 60min. Symbol "△" and "*" corresponds the peak positions of PbI_2

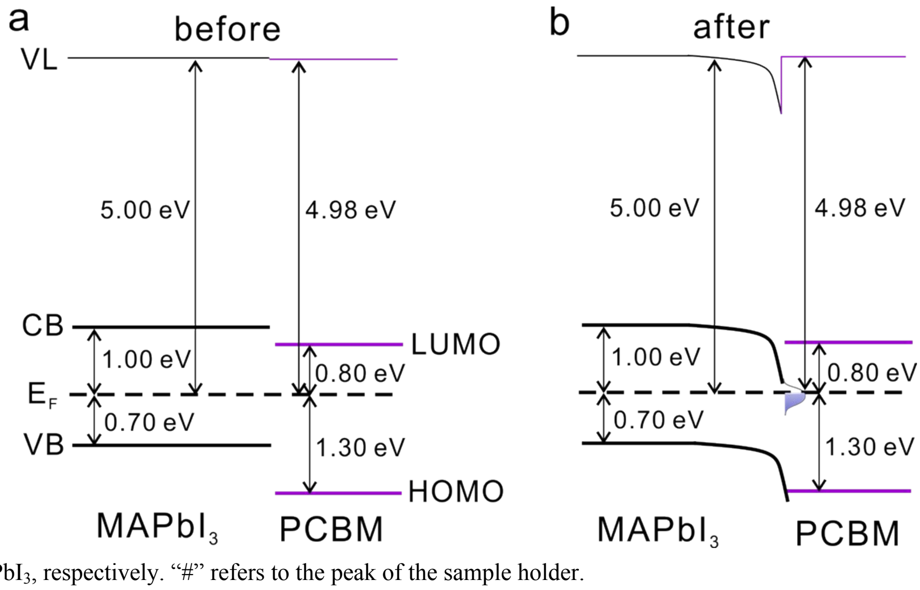

and $MAPbI_3$, respectively. "#" refers to the peak of the sample holder.

Fig S9. The energy level diagrams at the $MAPbI_3$/PCBM interface before and after illumination treatment in UHV condition for 60 min. Electronic bandgaps of 1.70 eV for $MAPbI_3$[3] and of 2.1 eV for PCBM[4] are assumed according

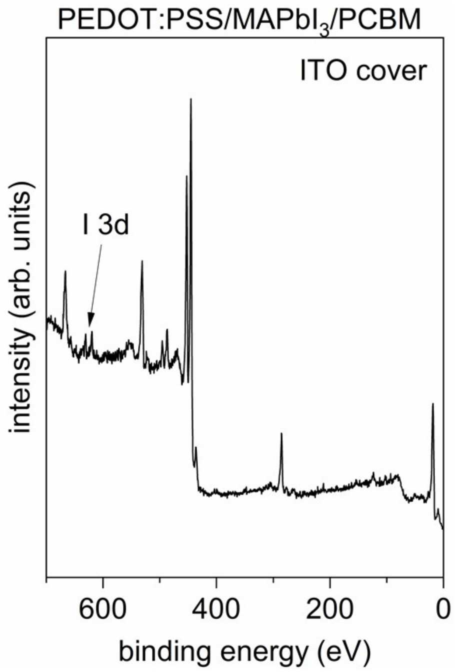

to previous reports. CB, VB and VL denote conduction bands, valence bands and vacuum level, respectively. LUMO and HOMO stand for lowest unoccupied and highest occupied molecular orbitals, respectively. E_F refers to Fermi level at 0 binding energy.

Fig S10. XPS survey spectrum of the ITO cover mounted on top of the PEDOT:PSS/$MAPbI_3$/PCBM film during light exposure in UHV condition. Iodine 3d peaks are indicated.

XRD patterns of the $MAPbI_3$ perovskite films prepared at the MAI/PbI_2 molar ratios of 1:1 and 1:0.95 are shown in Fig. S11. Clearly, the perovskite sample using the 1:1 ratio exhibits noticeable PbI_2 species in the film, as illustrated by the PbI_2 (001) peak at 12.7°. In comparison, by reducing the PbI_2 content by 0.05 percent, the formation of PbI_2 can be prevented.

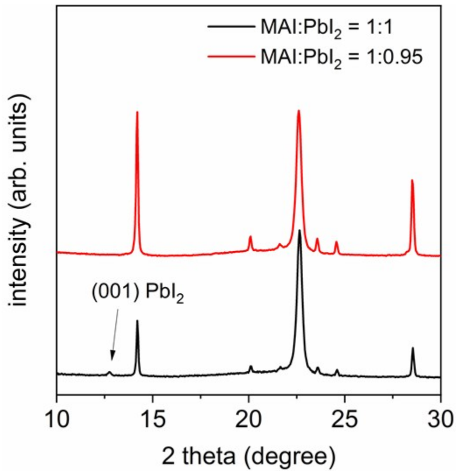

Fig. S11 XRD patterns of the $MAPbI_3$ perovskite films prepared at the MAI/PbI_2 molar ratios of 1:1 and 1:0.95.

References

[1] S. Wang, Y. Jiang, Emilio J. Juarez-Perez, Luis K. Ono, and Y. Qi, "Accelerated degradation of methylammonium lead iodide perovskites induced by exposure to iodine vapour", *Nature Energy* **2** (2016).

[2] M.-C. Jung, Y. M. Lee, H.-K. Lee, J. Park, S. R. Raga, L. K. Ono, S. Wang, M. R. Leyden, B. D. Yu, S. Hong, and Y. Qi, "The presence of CH3NH2 neutral species in organometal halide perovskite films", *Applied Physics Letters* **108**, 073901 (2016).

[3] P. Schulz, E. Edri, S. Kirmayer, G. Hodes, D. Cahen, and A. Kahn, "Interface energetics in organo-metal halide perovskite-based photovoltaic cells", *Energy & Environmental Science* **7**, 1377 (2014).

[4] K. Akaike, K. Kanai, H. Yoshida, J. y. Tsutsumi, T. Nishi, N. Sato, Y. Ouchi, and K. Seki, "Ultraviolet photoelectron spectroscopy and inverse photoemission spectroscopy of [6,6]-phenyl-C61-butyric acid methyl ester in gas and solid phases", *Journal of Applied Physics* **104**, 023710 (2008).
